# Wirelessly Controlled Implantable System for On-demand and Pulsatile Insulin Administration

**DOI:** 10.1038/s41598-019-41430-8

**Published:** 2019-03-21

**Authors:** Seung Ho Lee, Joong Woo Ahn, Yong Chan Cho, Se-Na Kim, Cheol Lee, Gi Won Ku, Young Bin Choy, Hee Chan Kim

**Affiliations:** 1 Institute of Medical and Biological Engineering, Medical Research Center, Seoul National University, Seoul, 03080 Republic of Korea; 2 Seoul National University Hospital Biomedical Research Institute, Seoul, 03082 South Korea; 3 Interdisciplinary Program in Bioengineering, College of Engineering, Seoul National University, Seoul, 08826 Republic of Korea; 4 Department of Pathology, Seoul National University College of Medicine, Seoul, 03080 Republic of Korea; 5 Department of Biomedical Engineering, Seoul National University College of Medicine, Seoul, 03080 Republic of Korea

## Abstract

We propose a wirelessly controlled implantable system for on-demand and pulsatile insulin delivery with a more convenient and safer strategy than currently available strategies. The system is a combined entity of a magnetically driven pump (i.e., an MDP), external control device (i.e., an ECD) and mobile app. The MDP for implantation consists of a plunger, barrel and drug reservoir, where an accurate amount of insulin can be infused in a pulsatile manner only at the time when a magnetic force is applied to actuate the plunger in the barrel. The ECD at the outside body can modulate the MDP actuation with an electromagnet and its control circuit, and this modulation can be wirelessly controlled by the mobile app. As a safety feature, the mobile app is programmed to pre-set the restrictions for the insulin dose and administration schedule to avoid overdose. The system is shown to infuse insulin in a highly reproducible manner, but it does not allow for insulin infusion when the pre-set restrictions are violated. When tested with diabetic rats, the profiles of insulin plasma concentration and blood glucose level are similar to those of animals treated with a subcutaneous injection of the same dose of insulin.

## Introduction

Diabetes mellitus, in which the blood glucose level is chronically deregulated, is a complex disease affecting more than 410 million people worldwide^1^. For treatment, intensive insulin therapy has been widely accepted to attain euglycaemia and minimize hyperglycaemia at the desired times^2^. In clinical settings, therefore, multiple daily injections (MDIs) or continuous subcutaneous insulin infusion (CSII) have been prescribed depending on each individual patient’s need, which has been reported to achieve satisfactory glycaemic control to a large extent^3^. However, as diabetes is a chronic disease, such a complex regimen of insulin administration should be performed throughout the lifespan; therefore, this treatment approach is somewhat invasive and painful, resulting in poor patient compliance. MDIs require at least three injections per day according to meal schedules^4^, and CSII pumps need frequent replacement of the infusion set with the cannula and needle, and the infusion site is recommended to be changed every few days^5^.

In this aspect, implantable devices for insulin infusion have gained extensive attention, where the devices are designed to deliver insulin in a minimally invasive, on-demand manner after one-time implantation^6–11^. To allow for circadian, pulsatile insulin delivery, insulin infusion was mediated mostly by mechanical actuation. For this purpose, many previous devices were equipped with electronic control circuits and power sources, i.e., batteries^6–10^. Because of the components required, the devices became somewhat large for implantation, and moreover, additional major surgery for device replacement was inevitable when the battery died. To resolve this issue, we previously introduced a magnetically driven pump (MDP) that could be implanted in the body for on-demand and pulsatile insulin infusion^12^. The actuation was performed via a magnetic force applied from the outside skin; thus, the implanted device did not need batteries or any other electronics.

However, our previous device (i.e., the MDP) still needs improvement for patient safety and convenience. The device was controlled by the manual application of an external magnet, where the dose of insulin depended on the number of device actuations. Therefore, with this device alone, it would not be easy for the patients to recognize the number of device actuations and to consistently monitor a total daily dose or the dose of consecutive administrations and their intervals, which could be critical for the proper management of blood glucose to avoid hyperglycaemia or hypoglycaemia. The absence of easy controllability, therefore, may impair patient adherence to insulin therapy^13,14^. In this sense, in addition to non-invasive insulin infusion after implantation, the device previously developed in our work can benefit further from a system designed for the accurate management of the insulin dose with a patient-friendly interface.

**Figure 1:**
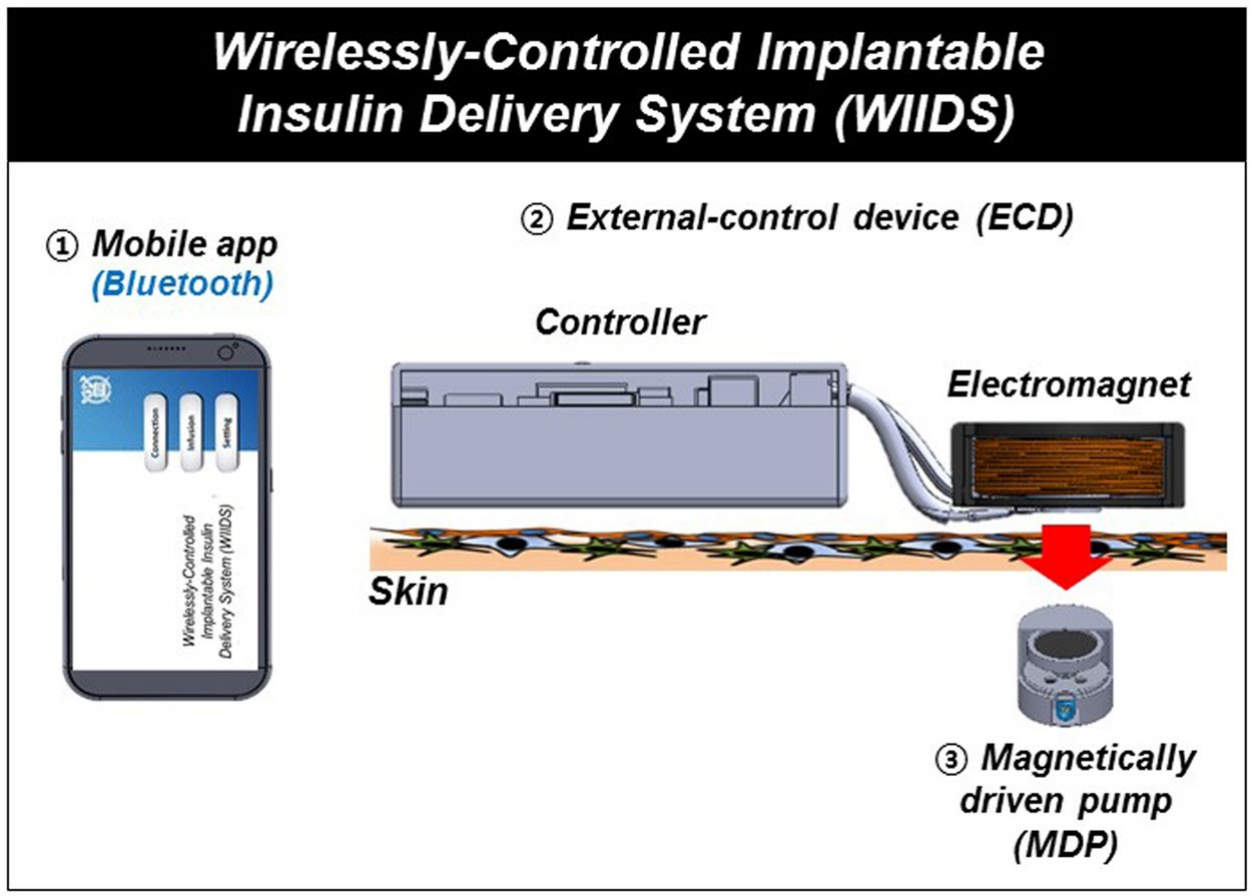
Schematic illustration of the wirelessly controlled implantable insulin delivery system (WIIDS). In the WIIDS, the implanted MDP was driven by the ECD in the outside body to infuse insulin, which was wirelessly controlled by the mobile app via Bluetooth.

Therefore, in this work, we propose a wirelessly controlled implantable insulin delivery system (WIIDS) as a combined entity of an implanted MDP and external control device (ECD), as well as an app for mobile devices, for non-invasive and on-demand insulin administration (Fig. 1). The ECD herein was packaged with an electromagnet and its controller, which could be located at the outside skin over the implanted MDP. Thus, only at the time when the insulin infusion was needed, the controller turned on the electromagnet, which in turn, actuated the MDP to infuse insulin. More importantly, we developed a mobile app that could generate the commands to modulate the ECD to allow for self-administration of insulin in a patient-friendly manner. The app contained a built-in safety function to prevent any possible scenarios of inappropriate insulin administration, hence preventing hypoglycaemic shock. In this work, the communications among the app, ECD and MDP were based on wireless means: the app can deliver the commands to the ECD via Bluetooth and then, the ECD located at the outside skin operates the implanted MDP via magnetic fields. In this way, the delivery profiles of insulin with versatile programming functions could be remotely controlled; thus, the system could provide a point-of-care monitoring strategy with improved patient compliance^15^.

To demonstrate the feasibility, we tested the system herein under both *in vitro* and *in vivo* environments, where the MDP was modulated via the ECD and app with different delivery scenarios. For *in vitro* evaluation, the MDP was immersed in pH 7.4 phosphate-buffered saline (PBS) at 37 °C underneath the ECD. For *in vivo* evaluation, we subcutaneously implanted the MDP and located the ECD on the outside skin, using streptozotocin (STZ)-induced diabetic rats, where the insulin and glucose levels in the blood were assessed. At the endpoint of the *in vivo* experiments, the tissue around the MDP was biopsied and analysed histologically for a safety evaluation.

## Results

### MDP design

**Figure 2:**
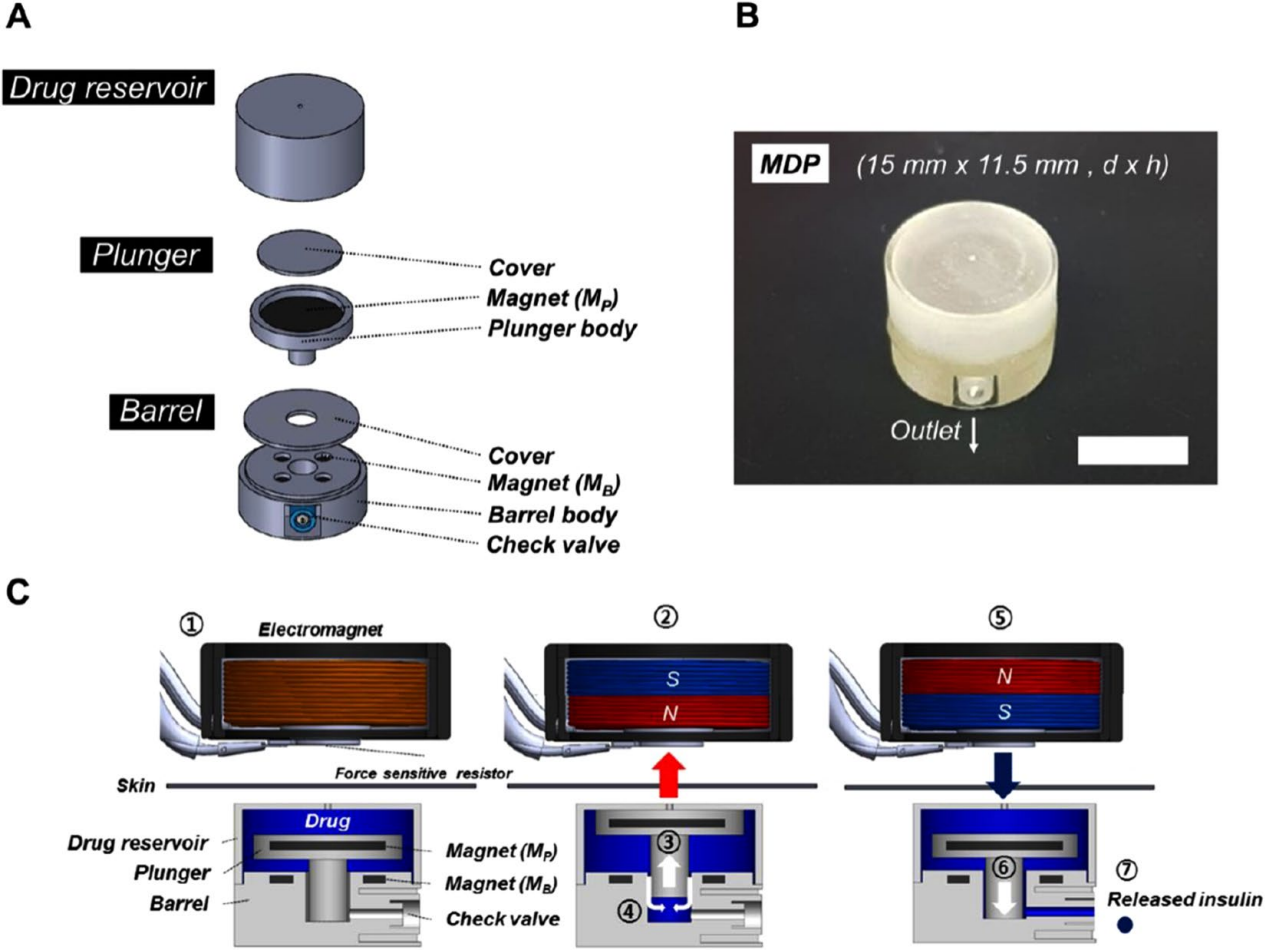
Design and operation principles of the magnetically driven pump (MDP). (**A**) 3D schematic images of the constituent units. (**B**) Optical image of the MDP. The scale bar is 1 cm. (**C**) Schematic description of the MDP operation. ① The electromagnet is placed above the implanted MDP; ② a positive voltage is applied, and the polarity of the electromagnet attracts the Mp; ③ the plunger moves upward, and ④ the insulin solution in the drug reservoir is aspirated into the barrel; ⑤ a negative voltage is applied, and the polarity of the electromagnet repels the Mp; ⑥ the plunger moves downward, and ⑦ the insulin is infused through the check valve.

The MDP for implantation was built based on the actuation principle proposed in our previous work^12^. Thus, the MDP was composed of three distinct constituent units, which were a drug reservoir, plunger and barrel, as shown in Figs 2A,B. A detailed fabrication procedure is described in Supplementary Fig. S1. In this work, the MDP was designed to with dimensions of 15 mm in diameter and 11.5 mm in height, with a total volume of approximately 2 ml, which was filled with 0.5 ml of an insulin solution (100 U ml^−1^). The entire surface of the MDP was coated with a biocompatible material, Parylene C^16^. In the MDP, the plunger and barrel were equipped with magnets, which faced each other with opposite polarities to attract each other; hence, there was no movement of the plunger and barrel. Therefore, the actuation principle of the MDP in this work was as follows (Fig. 2C). When an external magnetic field was applied to pull the plunger, the insulin solution in the drug reservoir was aspirated into the barrel. After that, an external magnetic field with the opposite polarity was applied to push the plunger and infuse the insulin solution in the barrel through the outlet port. At the outlet port, a check valve (2.0 mm Duckbill, Minivalve, USA) was installed to prevent the entrance of the external fluid during actuation.

### ECD design

**Figure 3:**
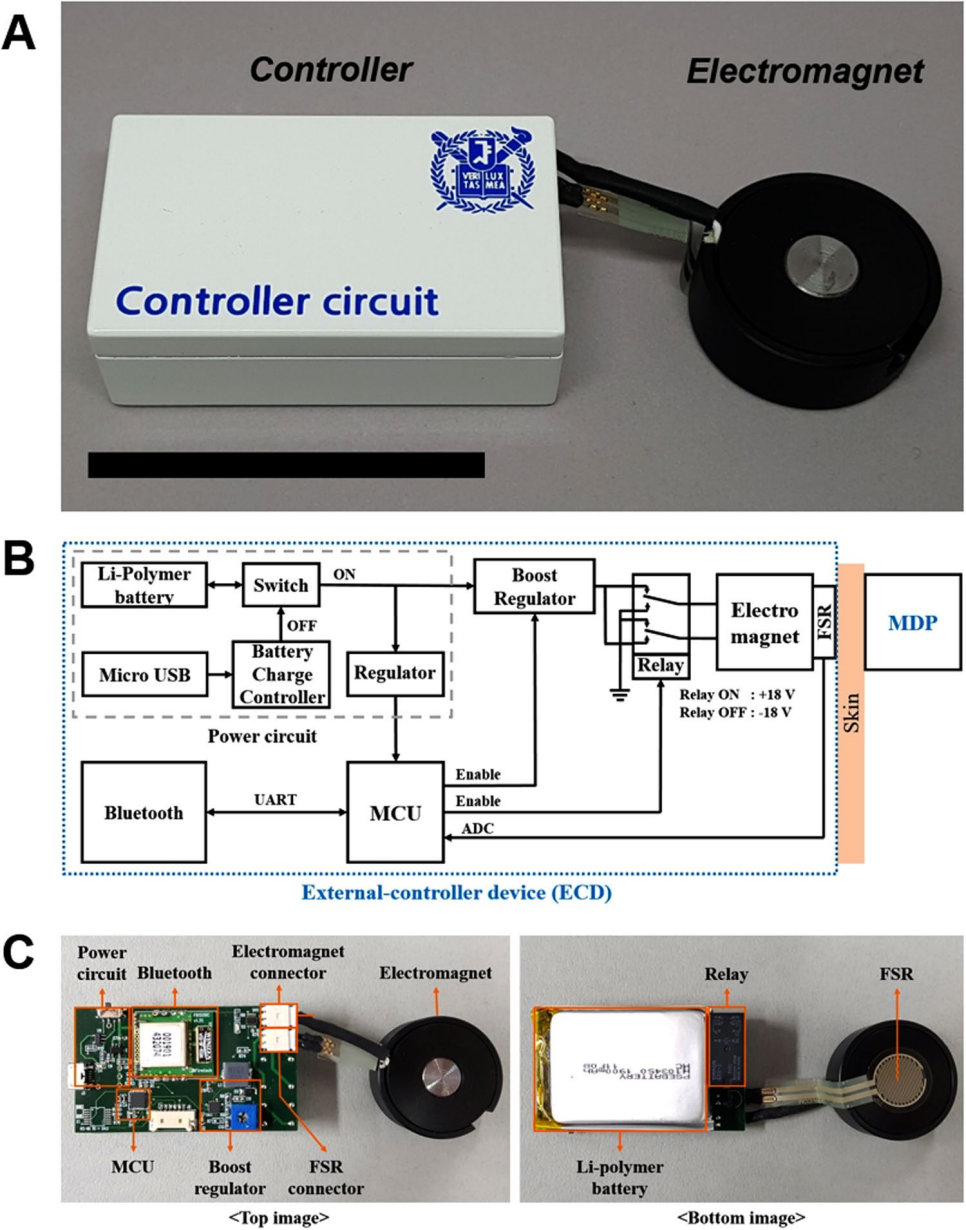
Development of the external control device (ECD). (**A**) Optical image of the ECD after packaging. The scale bar represents 5 cm. (**B**) System architecture block diagram of the controller circuit. **(C)** Optical images of the printed circuit board and electromagnet.

The ECD was designed to be located at the outside skin and was composed mainly of two distinct parts, namely, a controller and an electromagnet, as shown in Fig. 3A. As a prototype, the controller had dimensions of 65 × 35 × 18 mm^3^ (l × w × h), weighing 66 g, and an electronic circuit was built to turn the electromagnet on and off and to communicate with a mobile app wirelessly. The electromagnet, 30 mm in diameter and 12.5 mm in height, was custom-made with a cylinder of pure iron, 10 mm in diameter and 12.5 mm in height at the core, which was wound 800 times with a polyamide-enamelled copper wire, 0.25 mm in diameter (AIW, Taihan Electric Wire, Korea).

Figure 3B,C show the system block diagram and the printed circuit board with each of the components in the ECD. In this work, we attempted to generate a magnetic field strong enough to overcome the attraction force between the two magnets in the plunger and barrel in the MDP, which was measured to be 0.210 N (Advanced Force Measurement 9830, Interface, USA). For this purpose, a booster regulator (TPS55340-EP, Texas Instruments, USA) was designed to generate 18 V with a current of approximately 300 mA to the electromagnet. Under this condition, a magnetic field of about 0.5 T could be produced, which was known to propagate the tissues without damage^17^. This magnetic field was then able to generate a magnetic force of 0.292 N between the electromagnet and the magnet in the plunger. This force could be obtained for a gap of 4.5 mm between the two afore-mentioned units, considering the dimension of the MDP as well as the skin thickness of the animal models used in this work (~1 mm)^18^. However, the MDP herein could be still actuated at a gap of up to 2.5 mm, which was known to be the average skin thickness of humans^19^. To infuse insulin from the MDP, the electromagnet pulled and pushed the plunger by changing the magnetic polarity, respectively, and for this purpose, the polarity of the voltage applied to the electromagnet was changed by using a double-pole double-throw relay (G5V-2, Omron Electronics, Japan): the positive and negative voltages were applied when the relay was turned on and off, respectively. A force-sensitive resistor (FSR) was mounted at the bottom of the electromagnet to detect the mechanical actuation of the MDP, hence ensuring insulin infusion. The change in resistance was read through the change in voltage at the FSR, where two distinct waveforms, i.e., positive and negative square waveforms, were obtained as the plunger moved up and down during actuation, respectively. A microcontroller unit (MCU) controlled the boost regulator and the relay and converted the analogue signals from the FSR to digital signals. The commands for insulin infusion and the FSR data were wirelessly transmitted via a mobile app using Bluetooth (FB155BC, Firmtech, Korea). The electric power for the ECD was supplied by a 1-cell Li-polymer battery (H103450, Power Source Energy, China) with a capacity of 1900 mAh, which could be recharged via the micro USB.

### Mobile app design

**Figure 4:**
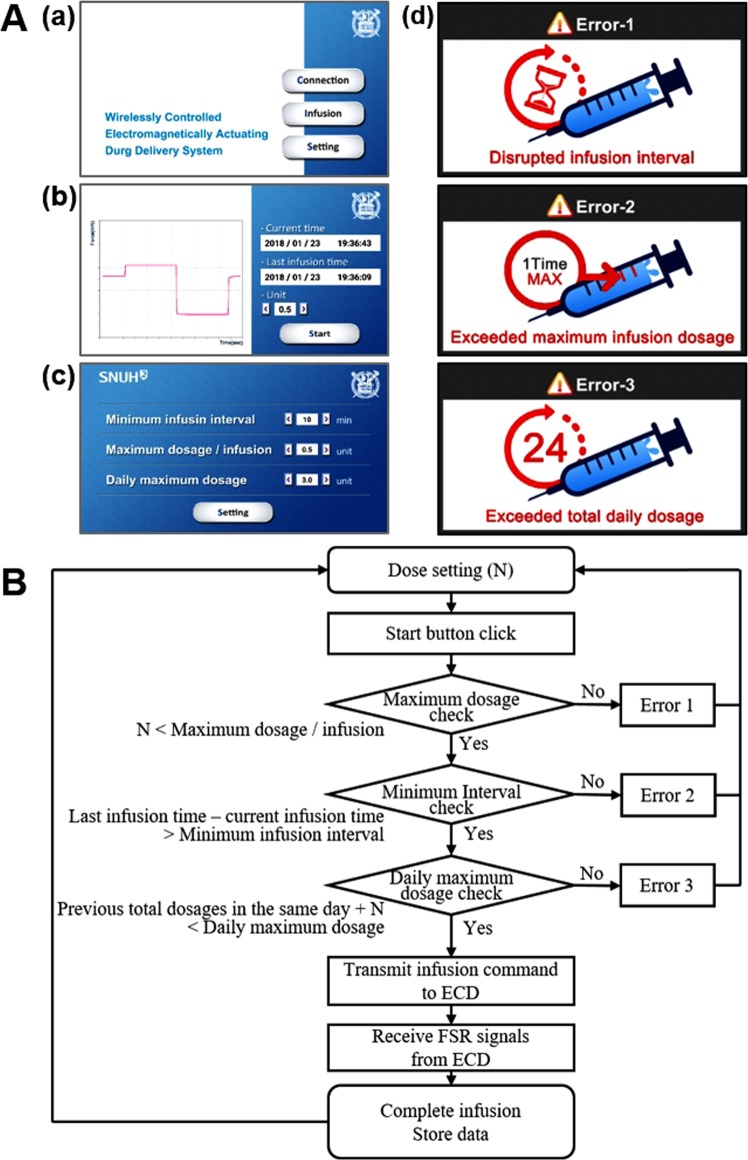
Description of the mobile app used to control the ECD. (**A**) Screenshots of the app embedded in a mobile device: (a) main screen with Bluetooth connection, insulin infusion and setting buttons; (b) screen for insulin infusion with a ‘Start’ button, also showing the information relevant to the insulin administrations; (c) screen for settings to manage the pre-set criteria of safety features; and (d) screens of three distinct errors due to the violation of each of the corresponding pre-set criteria. (**B**) Logic flowchart of the mobile app program.

To control the ECD in a patient-friendly manner, we also developed an Android-based mobile app with a graphical user interface (GUI), as shown in Fig. 4A. In the main screen (Fig. 4A(a)), the ‘Connection’ button activated the pairing of the mobile device and the Bluetooth in the ECD. The ‘Infusion’ button was then allowed to open a new screen (Fig. 4A(b)), displaying the ‘Start’ button, the current and last insulin-infusion times, and a text box for insulin dose selection, as well as a graph showing the voltage signals from the FSR. Thus, after dose selection, the ‘Start’ button could be pressed for the app to transmit the infusion command to the ECD, which in turn, applied the voltages to the electromagnet via the relay, as described above. In this work, two different insulin doses per administration, i.e., 0.5 and 1 U, were simulated, where the voltages to the electromagnet were generated once and twice, respectively, to actuate the MDP accordingly. During actuation, the app also received, displayed and stored the FSR digital signals in the mobile device to confirm the MDP actuations.

In the main screen (Fig. 4A(a)), the ‘Setting’ button opened another new screen (Fig. 4A(c)), where the safety feature needed for insulin delivery could be set up. In this work, therefore, the command from the app could be processed only when three pre-set criteria were met concurrently: (1) the minimum infusion interval (i.e., the minimum interval allowed between two consecutive administrations), (2) the maximum dosage/infusion (i.e., the maximum dosage allowed for each administration) and (3) the daily maximum dosage (i.e., the maximum dosage allowed for a day). Figure 4B shows the logic flow chart for the proposed system described herein. Thus, when any one of those criteria was not satisfied and the ‘Start’ button was pressed, the command could not be processed further, and the app displayed the corresponding error screen, as shown in Fig. 4A(d). Those limiting criteria could be set from the text boxes in the screen shown in Fig. 4A(c). In this work, the selection values for those criteria were set mainly to avoid hypoglycaemia and thus were listed based on reports from the previous clinical studies^20^. Thus, the minimum infusion intervals could be set at 10–600 min with an increment of 10 min; the maximum dosages/infusion could be set at 0.5 or 1 U; and the daily maximum dosages could be set at 0.5–3 U in increments of 0.5 U.

### *In vitro* performance test

**Figure 5:**
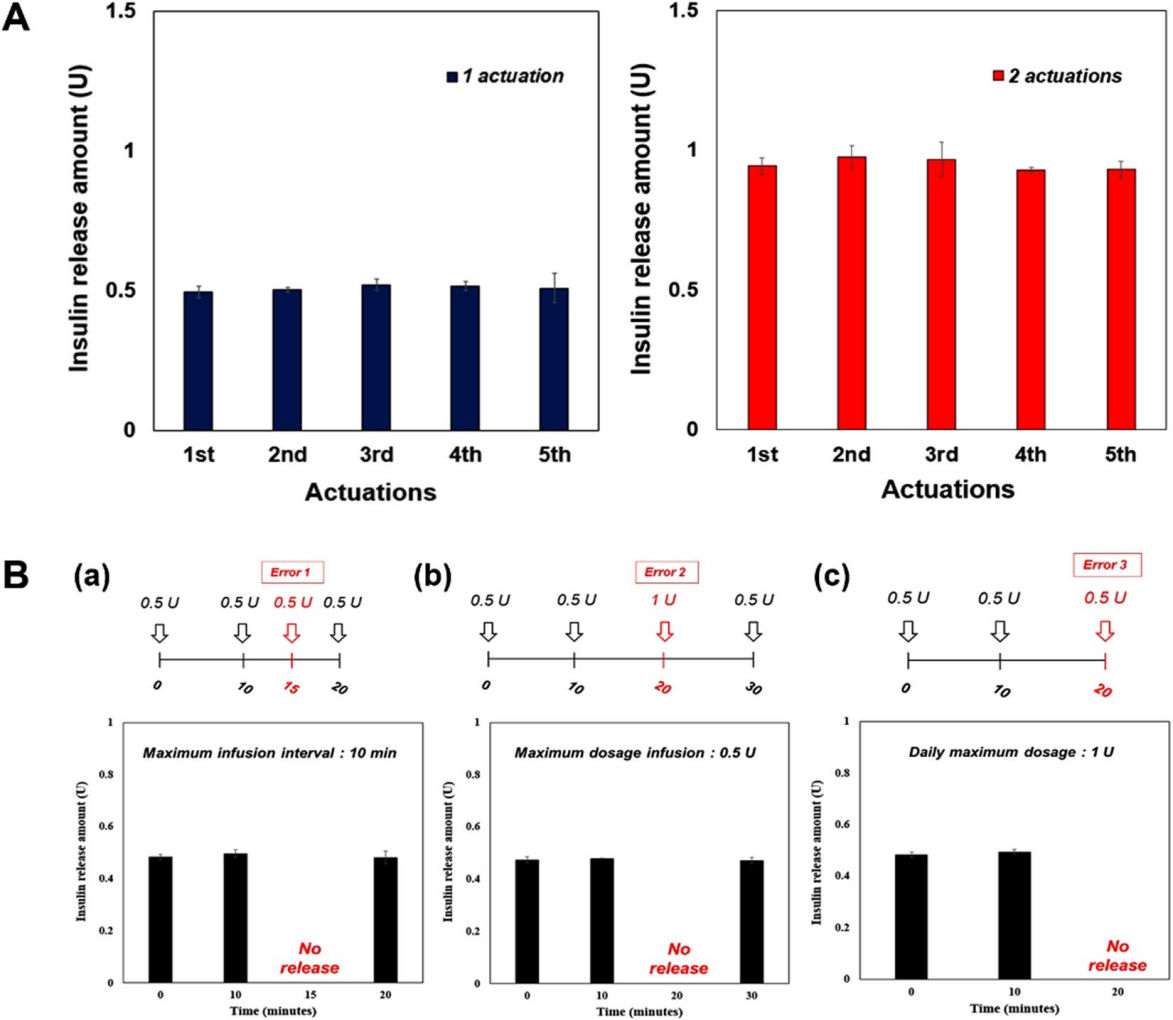
*In vitro* insulin infusion profiles of the WIIDS. (**A**) The MDP could infuse insulin with the WIIDS in a highly reproducible manner. The infused amounts of insulin were 0.509 ± 0.008 U and 0.948 ± 0.019 U with one and two consecutive actuations, respectively. The error bars are the s.d. (**B**) Insulin infusion could be restricted depending on the pre-set criteria of the safety features: (a) Error 1, due to a disrupted minimum infusion interval; (b) Error 2, due to an exceeded maximum infusion dosage; and (c) Error 3, due to an exceeded daily maximum dosage. The error bars are the s.d.

To evaluate the performance, the system herein was tested under *in vitro* environments, where the MDP was actuated while being fully immersed in the medium of pH 7.4 phosphate-buffered saline (PBS) at 37 °C. The ECD was located outside of the medium, with the electromagnet placed at 1 mm above the MDP to simulate the presence of skin after implantation^18^. A magnetic field was not expected to attenuate significantly, while propagating through the skin of this thickness^21^. For this specific performance test, the error settings were turned off just to assess the feasibility of insulin infusion with a combined system of the MDP and ECD. As shown in Fig. 5A, insulin was indeed infused into the medium when the MDP was actuated using the ECD. The amount of insulin infusion per actuation was highly reproducible, which increased proportionally with the number of actuations. In this work, for the settings of insulin doses at 0.5 and 1 U, the actual amounts of infused insulin were measured to be 0.509 ± 0.008 U and 0.948 ± 0.019 U with one and two actuations of the MDP, respectively.

To assess the safety features embedded in the ECD, we intentionally generated the commands to violate each of the three different error scenarios (Fig. 4A). To test Error-1, i.e., a disrupted minimum infusion interval, we set the allowable minimum infusion interval at 10 min and attempted the insulin infusions of 0.5 U at 0, 10, 15 and 20 min. As shown in Fig. 5B(a), insulin was observed to be infused at 0, 10 and 20 min; however, at 15 min, there was no infusion of insulin, as the interval (5 min) between the two consecutive commands was less than 10 min, and thus the app displayed the screen for Error-1 (Fig. 4A(d)) in the mobile device. For the test of Error-2, i.e., an exceeded maximum infusion dosage, we set the allowable maximum dosage per infusion at 0.5 U, and four consecutive commands for insulin infusion were generated in the order of doses of 0.5, 0.5, 1 and 0.5 U insulin. The interval between the two consecutive commands was maintained at 10 min in order to not violate the pre-set Error-1 scenario. Under this condition, insulin infusion was seen to be allowed at the dose of 0.5 U, while no insulin infusion was detected at the dose of 1 U, as shown in Fig. 5B(b). For this violation, we observed the appearance of the Error-2 screen on the mobile device (Fig. 4A(d)). To test Error-3, we set the allowable daily maximum dosage at 1 U. Then, infusion commands for a dose of 0.5 U were continuously generated at an interval of 10 min in order to not violate either of the pre-set scenarios for Error-1 and Error-2. As shown in Fig. 5B(c), insulin could be infused to a total dose of 1 U; however, from the third command, insulin was not infused, as the total dose set at 1 U would be exceeded. Again, the app displayed the screen for Error-3 in the mobile device (Fig. 4A(d)).

### *In vivo* evaluation

**Figure 6:**
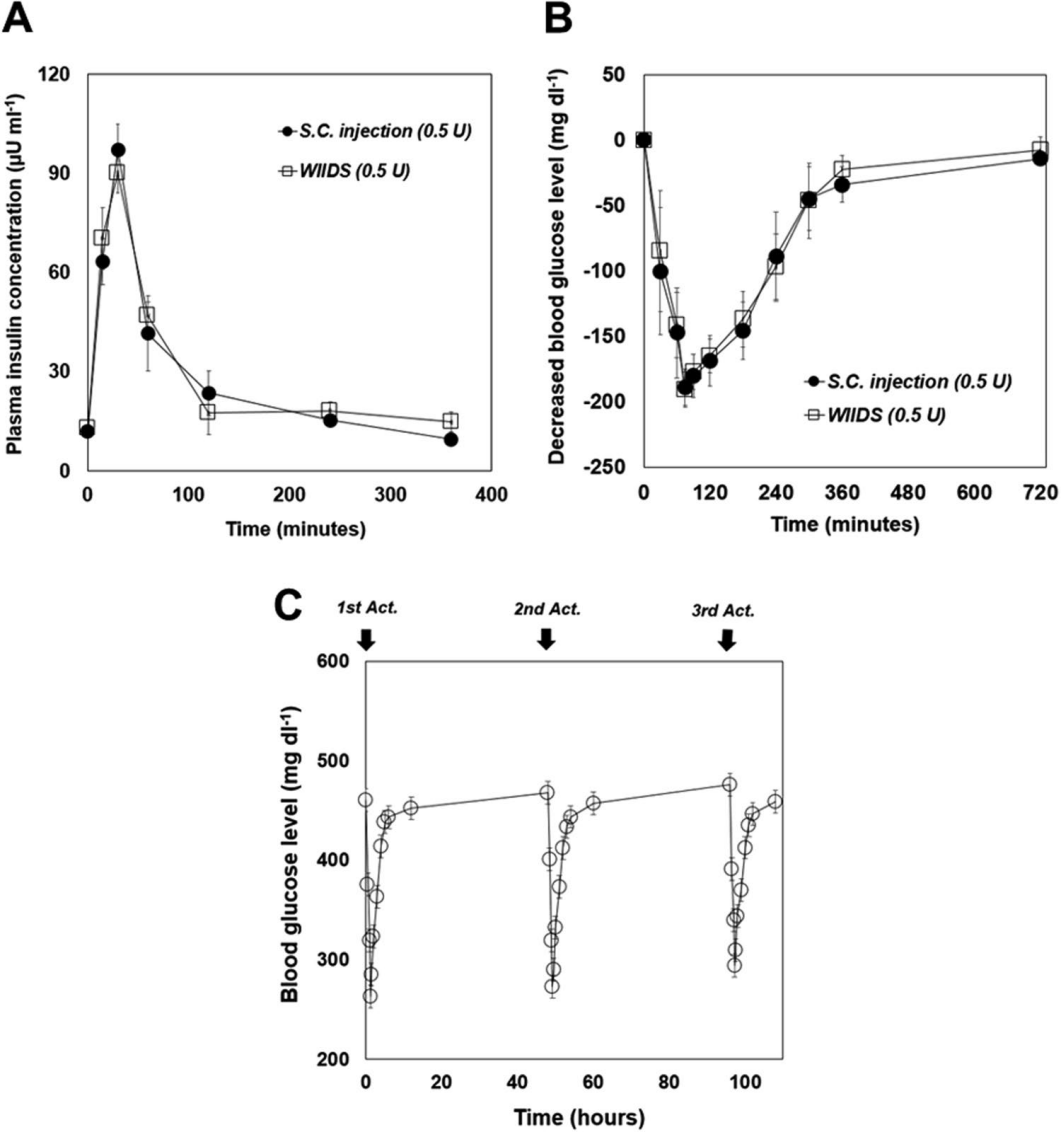
*In vivo* insulin infusion profiles of the WIIDS. (**A**) Profile of plasma insulin concentrations at -1, 15, 30, 60, 120, 240 and 360 min after insulin administration. The maximum insulin concentration occurred at 30 min in both the S.C. injection (n = 4) and WIIDS (n = 4) groups. The error bars are the s.d. (**B**) Profile of blood glucose levels at -1, 30, 60, 75, 90, 120, 180, 240, 300, 360, and 720 min after insulin administration. The maximum decrease in glucose level occurred at 75 min in both the S.C. injection (n = 4) and WIIDS (n = 4) groups. The error bars are the s.d. (**C**) Profile of blood glucose levels with multiple actuations of the MDP in the WIIDS (n = 3) group. The error bars are the s.d.

To examine the *in vivo* feasibility, we employed streptozotocin (STZ)-induced diabetic rats and administered insulin via the WIIDS and conventional subcutaneous injection to compare the pharmacokinetic/pharmacodynamic profiles of these two modalities. Thus, for the animals treated with the WIIDS (i.e., the WIIDS group), the MDP was subcutaneously implanted and actuated once with the ECD to administer 0.5 U insulin. For the diabetic rats treated with subcutaneous injection (i.e., the S.C. injection group), the same dose of insulin was injected subcutaneously. As shown in Fig. 6A, the pharmacokinetic profiles of insulin were similar between the WIIDS and S.C. injection groups, indicating that the MDP after implantation could be properly actuated with the ECD to infuse the insulin solution with the expected dose of 0.5 U. Thus, the C_max_ were obtained to be 90.2 ± 6.24 µU ml^−1^ and 97.2 ± 7.86 µU ml^−1^ for the WIIDS and S.C. injection groups, respectively, both of which were observed at T_max_ = 30 min. Because of this similarity, the pharmacodynamic profiles between the WIIDS and S.C. injection groups were also similar, showing minimum glucose levels of –190.7 ± 13.6 mg dl^−1^ and –189.3 ± 13.9 mg dl^−1^, respectively, at 75 min (Fig. 6B). To assess the feasibility of multiple insulin administrations, the WIIDS was actuated three times at intervals of 36 h. As shown in Fig. 6C, the profiles of the blood glucose levels were similar for all three actuations, suggesting that the WIIDS operations were reproducible under the *in vivo* testing environments in this work. The interval between insulin administrations was set for practical reasons in animal health as the blood could not be retracted very often. For this reason, the overall blood glucose levels could not be controlled perfectly with the diabetic animals treated *ad libitum*. However, the WIIDS herein was still shown to infuse the needed dose of insulin only at the times when the command was delivered from the mobile app. Our previous work also showed that the MDP could deliver an accurate dose of insulin reproducibly for a longer period of 60 days^12^.

### Histopathologic evaluation

To confirm the *in vivo* biocompatibility, biopsied tissues around the MDP were obtained at the end of the experiments (7 days), and they were stained with H&E and examined by a pathologist. As shown in Supplementary Fig. S2, the overall inflammatory response and foreign body reactions were observed to be minor. The MDP herein was hermetically sealed and thus, only the outer surface of the MDP was in contact with the tissues after implantation. Therefore, the biocompatibility of the MDP could be ascribed to the coating material, Parylene C, used in this study^16^. This result could be further supported by a long-term biocompatibility of the Parylene C-coated MDP in our previous work^12^. The tissues surrounding the MDP exhibited fibrous capsules composed of fibroblasts, collagen, and capillaries; however, this finding was also reported in many previous studies with nondegradable implanted devices.

## Discussion

In this work, we developed a WIIDS, a combined entity of a batteryless, implantable device and its wireless control unit, to propose a more convenient and safer strategy for minimally invasive and on-demand insulin infusion. For the implanted device, we employed an MDP, which could be actuated noninvasively via an externally applied magnetic field without a battery or control circuit inside^12,22^. Although this device could be operated with a magnet outside the body, it may not be easy to control and monitor the insulin dose or regimen conveniently according to the perspectives of patients. Monitoring and controlling insulin easily could be also very important, considering that improper insulin administration could cause a fatal condition, such as hypoglycaemia^23^.

Therefore, we proposed the ECD and mobile app to provide more accurate control over insulin doses and administration schedules, hence improving safety. Given the simple actuation principle of the MDP, the ECD could generate a DC voltage to turn on the electromagnet and actuate the MDP only at the time when the infusion was needed. Unlike the MDP in our previous study, where insulin infusion depended mainly on the attraction force between the magnets in the plunger and barrel, the ECD herein could easily reverse the polarity of magnetic field to actually push the plunger (Fig. 2C), thereby providing more obvious insulin infusion. Given this, multiple actuations of the MDP were also possible, showing a reproducible dose increase (Fig. 5A). To ensure the infusion further, the FSR was embedded in the ECD to allow for the detection of the pull-and-push movement of the plunger in this work (Fig. 4A(b)).

The ECD was also equipped with Bluetooth, allowing wireless transmission of the commands and data between the ECD and the mobile device. Due to this feature, a software application in a mobile device, i.e., a mobile app, was able to control the whole scenario of insulin administration (Figs 4 and 5). Therefore, the app could block the cases from causing hypoglycaemia without exception by not allowing the commands to be transmitted (Fig. 5B). In addition, the history of insulin administration, along with the FSR data, could be wirelessly transmitted and stored in a mobile device, which was enabled with the app developed in this work. Therefore, the management of diabetes with such a mobile app is expected to improve the quality of life of the patients, as reported in other previous clinical studies^24^.

In this work, the WIIDS was prepared as a proto-type to give insight into better control of insulin administration using a batteryless, implantable MDP, and thus, more considerations need to be made for its actual clinical application. The ECD in this work needs to be properly packaged to be worn on the outside of the body over the implanted MDP in a more convenient way, as done with a continuous insulin infusion system that has been proven to be acceptable for diabetic patients^25^. In this regard, it would be advantageous to improve the ECD to be equipped with a more highly integrated circuit^26^ and a lighter electromagnet^27,28^. In this study, we designed the MDP to infuse 0.5 U insulin per actuation mainly for prandial insulin administration in clinical settings^20^. However, to allow for better management of a more complex regimen including basal insulin administration, more versatile insulin doses would be useful, which could be realized by optimizing the actuation distance of the plunger or the insulin concentration in the drug reservoir of the MDP or by adding more sophisticated functions in the app.

In this work, the drug reservoir in the MDP was made to be small for its *in vivo* feasibility test with small animals. Therefore, considering a long-term human use, the MDP should be made larger to contain more insulin in the drug reservoir. For example, with the insulin concentration employed in this study and a typical high dose of prandial insulin (~6 U)^29^, the volume of the drug reservoir may increase to about 32 ml, which would allow for a 6-month use until a refilling procedure. However, the volume of the actuation part would not increase, as it would be still operable without any additional driving units and thus, the MDP could be still small compared with the other implantable drug-infusion pumps in clinical use (~100 ml)^30,31^. In our previous work, the MDP, when connected with a catheter, could still infuse an accurate dose of insulin^12^. In this aspect, the MDP in combined with the app and ECD is also expected to benefit from insulin delivery via the intraperitoneal route.

In conclusion, we have proposed the WIIDS for on-demand, pulsatile insulin delivery with safety and convenience features. The implanted MDP can be actuated with a non-invasive magnetic force applied from the ECD at the outside body. Therefore, an accurate amount of insulin can be infused in a highly reproducible manner only at the time when an electromagnet in the ECD is activated. The mobile app can control the ECD wirelessly via Bluetooth, allowing for on-demand insulin administrations. The app is also able to set up the criteria to limit insulin overdose, where the command for insulin infusion is configured not to be performed whenever the restrictions are violated. With those added benefits, the WIIDS herein can deliver insulin similarly to a conventional, subcutaneous injection. Therefore, we conclude that the WIIDS proposed in this work is a promising strategy for more patient-friendly and safer insulin delivery.

## Method

### *In vitro* insulin infusion study

The performance of the WIIDS was tested under *in vitro* environments, where the MDP was fully immersed in phosphate buffered saline (PBS; pH 7.4) at 37 °C and actuated with the ECD via a mobile app at scheduled times. During actuation, the gap between the MDP and the electromagnet was set at 1 mm to simulate the presence of skin after implantation^18^. The collected aliquot was analysed with a high-performance liquid chromatograph (Agilent 1260, USA) equipped with a UV detector set at 215 nm and a reversed-phase Diamonsil C_18_ column (5 μm, 150 mm × 4.6 mm i.d., Dikma, USA). The mobile phase consisted of 0.1% trifluoroacetic acid aqueous solution and acetonitrile (6:4, v v^−1^). The flow rate and column temperature were set at 1 ml min^−1^ and 20 °C, respectively^32^. All experiments were performed in triplicate for each of the scheduled actuations.

### *In vivo* experiments

The protocols for *in vivo* experiments were approved by the Institutional Animal Care and Use Committee at Seoul National University Hospital Biomedical Research Institute (IACUC No. 17–0016). All experiments were performed in accordance with the relevant guidelines and regulations. To prepare diabetic animal models, male Sprague-Dawley (SD) rats weighing 350–400 g were fasted for 8 h with free access to water and then, 60 mg kg^−1^ streptozotocin (STZ; Sigma-Aldrich, USA) was injected intraperitoneally to destroy the insulin-producing beta cells of the pancreas^33^. After 5 days, the rats were fasted for 8 h, and approximately 3 μl of blood was collected from the tail vein and measured with a glucometer (Accu-Chek® Performa, Roche Diagnostics, Germany). In this experiment, only rats with blood glucose levels above 300 mg dl^−1^ were used as diabetic rats.

For the WIIDS group, the MDP was implanted in a subcutaneous space (Supplementary Fig. S3) and actuated to infuse 0.5 U insulin using the ECD via a mobile app. For the S.C. injection group, the same dose of insulin solution was injected subcutaneously. To analyse the profile of plasma insulin concentration, 0.3 ml of blood was collected at −1, 15, 30, 60, 120, 240, and 360 min after insulin administration. Then, the blood plasma was separated by centrifugation and stored in the freezer at −20 °C before analysis. In this work, insulin concentration in plasma was measured using an ELISA kit (Mercodia Insulin ELISA, Mercodia AB, Sweden). To assess the profile of blood glucose level, blood was withdrawn and measured at −1, 30, 60, 120, 240, 360 and 720 min after insulin administration, using a glucometer (Accu-Chek Performa, Roche Diagnostics, Germany).

### Histopathology

For histopathologic evaluation, the rats of the WIIDS group were euthanized by carbon dioxide inhalation 7 days after implantation, and the tissues surrounding the implanted MDP were harvested. The resulting tissues were fixed with 4% paraformaldehyde for 24 h and embedded in paraffin wax. Subsequently, the paraffinized tissue samples were cut into slices 4 µm in thickness to prepare the tissue slides, which were then stained with haematoxylin and eosin (H&E)^34^. In this work, the stained slides were examined by a professional pathologist using an optical microscope (BX53, Olympus, Japan) (Supplementary Fig. S2). At least 3 images were obtained from each of the four rats, and thus, a total of 12 images were evaluated. The control images from the unwounded, normal tissues were not assessed as they would not provide with the useful information to be actually compared with.

### Statistical analysis

The data for the plasma insulin concentrations and blood glucose levels at each scheduled time point were compared between the S.C. injection and WIIDS groups for statistical significance via non-parametric methods (Kruskal-Wallis ANOVA). *P* < 0.05 was considered statistically significant. The statistical software application used was SPSS (SPSS version 22, IBM, USA).

## Supplementary information

Supplementary Information
