# Supplementary material for: Wirelessly Controlled Implantable System for On-demand and Pulsatile Insulin Administration
Source: Sci Rep. 2019 Mar 21;9:5009. doi: 10.1038/s41598-019-41430-8 (PMC6428930; doi:10.1038/s41598-019-41430-8)
Supplement: Supplementary file 1 — Supplementary Information [file 41598_2019_41430_MOESM1_ESM.docx]

**Wirelessly Controlled Implantable System for On-demand and Pulsatile Insulin Administration**

Seung Ho Lee^+^, Joong Woo Ahn^+^ ,Yong Chan Cho, Se-Na Kim, Cheol Lee, Gi Won Ku, Young Bin Choy*, Hee Chan Kim*


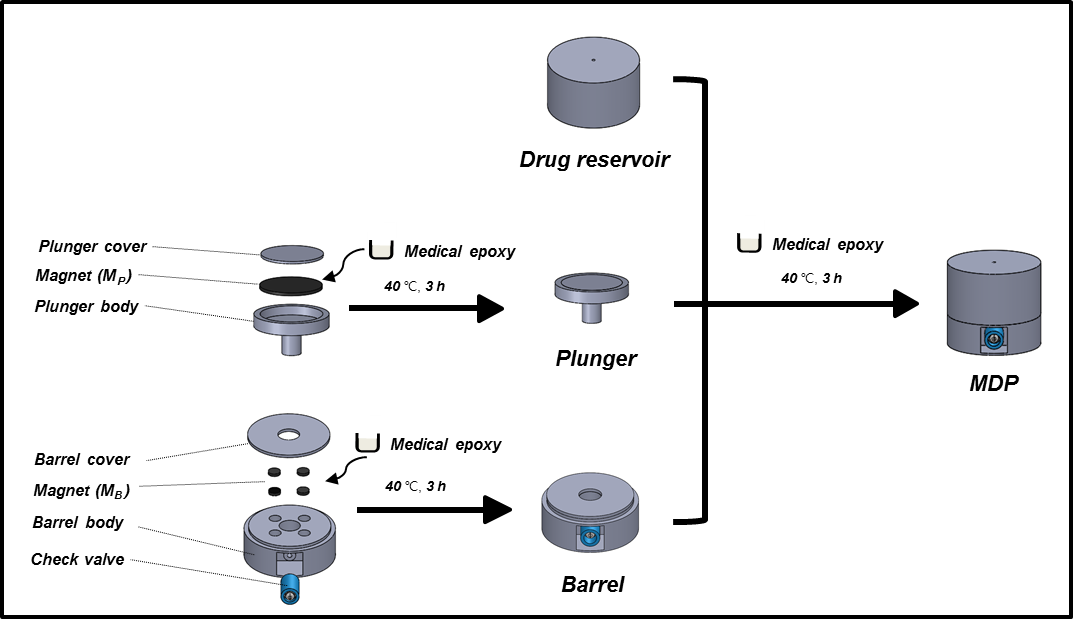


**Supplementary Figure S1. Detailed description of the MDP fabrication procedures.** Each of the parts was first designed and drawn using 3D modelling software (SolidWorks 2014, USA). Then, the units were each fabricated with a polyurethane copolymer (Fullcure® 720 photopolymer) using a 3D printer (Eden 350V, Objet Geometries, Israel). Then, the resulting parts were assembled with the magnets for the plunger and barrel (~ 0.1 T), which were attached with medical epoxy (Epo-Tek 301, Epoxy Technology, USA).


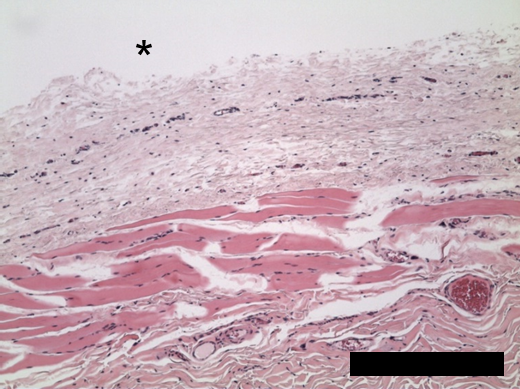


**Supplementary Figure S2.** Representative H&E-stained image of the biopsied tissues around the MDP obtained 7 days after implantation. The asterisk (*) indicates the location of the MDP. The scale bar represents 200 μm.


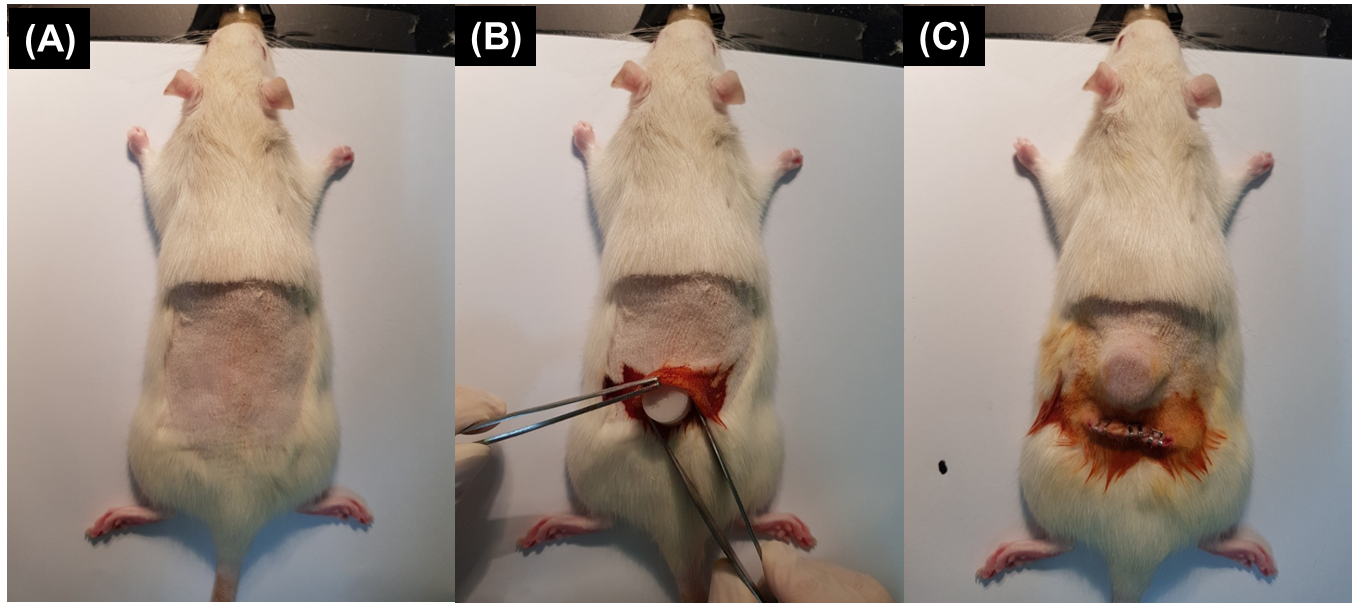


**Supplementary Figure S3. Surgical procedure for MDP implantation.** **(A)** A rat was anaesthetized with isoflurane, and the dorsal area was shaved. **(B)** After the area was sterilized with betadine, a skin incision (1-2 cm) was made, and the MDP was implanted into the subcutaneous pocket. **(C)** The wound was closed with a surgical clip and disinfected with betadine.
